# Social Determinants of Mental Health During a Year of the COVID-19 Pandemic

**DOI:** 10.1017/S0954579422000396

**Published:** 2022-07-07

**Authors:** Savannah Minihan, Amy Orben, Annabel Songco, Elaine Fox, Cecile D. Ladouceur, Louise Mewton, Michelle Moulds, Jennifer H. Pfeifer, Anne-Laura Van Harmelen, Susanne Schweizer

**Affiliations:** 1 University of New South Wales, Sydney, Australia; 2 MRC Cognition and Brain Sciences Unit, University of Cambridge, Cambridge, United Kingdom; 3 University of Oxford, Oxford, United Kingdom; 4 University of Pittsburgh, Pittsburgh, United States of America; 5 University of Oregon, Eugene, Oregon, United States of America; 6 Leiden University, Leiden, The Netherlands; 7 University of Cambridge, Department of Psychology, Cambridge, United Kingdom

**Keywords:** COVID-19, Physical distancing, Mental health, Social connectedness, Social rejection sensitivity

## Abstract

Belonging is a basic human need, with social isolation signaling a threat to biological fitness. Sensitivity to ostracism varies across individuals and the lifespan, peaking in adolescence. Government-imposed restrictions upon social interactions during COVID-19 may therefore be particularly detrimental to young people and those most sensitive to ostracism. Participants (*N* = 2367; 89.95%; female, 11-100 years) from three countries with differing levels of government restrictions (Australia, UK, and USA) were surveyed thrice at three-month intervals (May 2020 – April 2021). Young people, and those living under the tightest government restrictions, reported the worst mental health, with these inequalities in mental health remaining constant throughout the study period. Further dissection of these results revealed that young people high on social rejection sensitivity reported the most mental health problems at the final assessment. These findings help account for the greater impact of enforced social isolation on young people’s mental health, and open novel avenues for intervention.

The global COVID-19 pandemic brought about unprecedented disruptions to our daily lives. Governments imposed restrictions and limitations on social life, leading to considerable reductions of face-to-face social contact for people the world over. Involuntary curbs to social interactions conflict with humans’ basic need for belonging (Maslow, 1943). Psychologically and neurobiologically, humans interpret involuntary social isolation as social ostracism (Cacioppo & Cacioppo, 2016). Sensitivity to ostracism (Somerville, 2013) peaks in adolescence (10-24 years; Sawyer et al., 2018), a period of life when young people are biologically and socially primed to spend time with their peers (Lam et al., 2014). This developmental imperative for social reorientation has been disrupted by the pandemic. Adolescents may therefore be particularly vulnerable to adverse effects of these social restrictions on their mental well-being (Orben et al., 2020).

## Adolescent Mental Health During the COVID-19 Pandemic

Studies investigating the impact of the pandemic on adolescent mental health have provided evidence for its detrimental impact on this age group. Cross-sectional research comparing data collected during the pandemic to pre-pandemic statistics consistently demonstrates elevated levels of mental health problems in adolescents during the pandemic (Chen et al., 2020; Duan et al., 2020; Racine et al., 2020; Xie et al., 2020; Zhou et al., 2020). Longitudinal studies show similar yet somewhat more equivocal trends. While most longitudinal studies reported an increase in symptoms of depression and anxiety (Chahal et al., 2020; De France et al., 2021; Magson et al., 2020) during the pandemic, a set of white papers showed no changes in internalizing symptoms (Raw et al., 2021), or an increase in depression only, but not anxiety (Barendse et al., 2021). Barendse et al. (2021), a noteworthy study due to its large (*N* = 1,339) and international sample of 9-18-year-olds, found that the most negative mental health impacts were reported by adolescents under ‘lockdown’ restrictions. Together these findings suggest that young people indeed suffer. However, it remains unclear whether they are at greater risk of pandemic-related mental health problems than adults. The current study therefore examined age-related variance in mental health problems in a sample ranging from early adolescence to old age (11-100 years). Mental health problems were assessed at three time points, at three-month intervals during the first year of the pandemic from May 2020 to April 2021. To explore whether more stringent government restrictions to social interactions had a more detrimental impact on mental health during the pandemic, participants were included from the USA, UK and Australia; three countries that varied in their government restriction stringency indices (Hale et al., 2021; the stringency index captures a range of government restrictions such as school/work closures and stay-at-home orders). Importantly, the current study went beyond describing the extent of the mental health impact of COVID-19 and related government restrictions, by exploring why young people may be at greater risk for experiencing mental health problems during the pandemic.

## Why are Adolescents at Greater Risk for Mental Health Problems During the COVID-19 Pandemic?

Adolescence is a period of risk for the development of mental health disorders, with 75%; of all cases emerging by age 24 (Kessler et al., 2005). Compared to adults, adolescents experience more negative affect and more variable mood states in their everyday lives (Larson et al., 2002; Silvers et al., 2012). Such emotional reactivity occurs in the context of cognitive development and social change, marked by increased time spent with peers and heightened sensitivity to peer acceptance and influence (Blakemore & Mills, 2014; Burnett & Blakemore, 2009; Kilford et al., 2016). Adolescents, particularly girls (Benenson et al., 2013; Paquette & Underwood, 1999; Reijntjes et al., 2006; Rowe et al., 2015; Zimmer-Gembeck, 2015), are also hypersensitive to social rejection (Blakemore & Mills, 2014), with research showing that social rejection has a greater negative impact on adolescents’ mood, compared to adults (Sebastian et al., 2010). Given the social sensitivity that characterizes adolescents as well as their increased need for peer interaction (Orben et al., 2020), the physical distancing measures that have been imposed as a result of the COVID-19 pandemic may have had a greater negative impact on adolescents, compared to adults (Orben et al., 2020).

### Social Connectedness

Reduced social connectedness (Orben et al., 2020) due to physical distancing measures during the pandemic may be one determinant of poor mental health in young people (Hoffart et al., 2020). This hypothesis has been supported by findings from longitudinal studies. For example, greater increases in depression and anxiety symptoms (Magson et al., 2020) as well as suicidal ideation (Hutchinson et al., 2021) were observed in adolescents who felt socially disconnected, whereas 7-15-year-olds who reported more social connectedness were less likely to develop mental health problems after experiencing pandemic-related stressors (Rodman et al., 2021). In line with these findings, a systematic review on the impact of social isolation and loneliness on adolescent mental health in the context of COVID-19 indicated that they were likely to significantly increase the risk of mental health problems in young people (Loades et al., 2020).

Indeed, loneliness has long been considered a significant public health issue. It can impair executive functioning, sleep, as well as physical and mental well-being and lead to an increased risk of morbidity and mortality (Cacioppo & Cacioppo, 2014). High levels of loneliness in the context of COVID-19 have been reported, particularly in young people (Groarke et al., 2020; Killgore, Cloonan, Taylor, & Dailey, 2020; Li & Wang, 2020; Rosenberg et al., 2021). Social support in turn was found to be protective against increased loneliness (Groarke et al., 2020). Heightened loneliness and decreased social support during the pandemic were associated with elevated depression, anxiety, and suicidal ideation (Hoffart et al., 2020; Hutchinson et al., 2021; Iob et al., 2020; Killgore, Cloonan, Taylor, & Dailey, 2020; Killgore, Cloonan, Taylor, Miller, et al., 2020; Palgi et al., 2020; Rodman et al., 2021). However, the adverse effects of increased loneliness due to periods of enforced physical distancing may have been mitigated by engaging in social interactions with peers and family via online platforms. The potential of online social interactions for mitigating loneliness and social disconnection, however, remains under-researched. In a rare prospective study, participants who felt more lonely during the pandemic were less likely to seek out online interactions (Breen et al., 2020). In another study, youth who reported lower levels of digital socialization had heightened internalizing symptoms during the pandemic, controlling for pre-pandemic symptoms (Rodman et al., 2021), while in a cross-sectional study of young people, there was no association between the frequency of virtual social interactions and well-being (Towner et al., 2021). Loneliness, social support, and social interactions may therefore interact to contribute to changes in mental health throughout the pandemic. This may be particularly the case for adolescents, who show greater reliance on their peers for social connectedness.

Reduced social connectedness, we propose, is most detrimental to individuals with high levels of sensitivity to social rejection. Social rejection sensitivity refers to the tendency to anxiously expect, readily perceive, and overreact to social rejection (Downey & Feldman, 1996). Social rejection sensitivity has been linked both cross-sectionally and prospectively to depressive and anxiety symptoms (Gao et al., 2017). There is also evidence that rejection sensitivity is associated with an aversion to aloneness (Molinari et al., 2020). Molinari et al. suggested that individuals high in social rejection sensitivity, in particular, tend to see aloneness as something negative, an indicator of being rejected (Molinari et al., 2020). Indeed, as noted at the outset, as a species, involuntary social isolation signals rejection from the group and is associated with risk to biological fitness. Thus, reduced face-to-face social contact may be particularly detrimental for rejection sensitive individuals, as they are likely to perceive this reduced social contact as an indication of rejection.

### The Current Study

The current study longitudinally examined the relationship between social connectedness, social rejection sensitivity and mental health from early adolescence to old age (11-100 years) during the COVID-19 pandemic. Data were drawn from the COVID-19 Risks Across the Lifespan (CORAL) study, an online longitudinal study, comprising predominately female participants, designed to investigate how the changes brought about by the COVID-19 pandemic have affected people’s wellbeing and social connectedness. Baseline data (T1) were collected between May 5^th^, 2020 and September 30^th^, 2020. Participants who completed at least 65%; of the baseline survey were re-contacted via email to complete two follow-up measures (T2: August 5^th^, 2020 – January 29th 2021, T3: November 5^th^ 2020 – April 9th, 2021). Critically, the design of the study allowed for a naturalistic manipulation of the level of physical distancing and enforced social isolation experienced by participants. Specifically, by recruiting individuals from Australia, the UK, and USA, we were able to investigate the extent to which differing levels of government restrictions in response to COVID-19, including physical distancing, influenced our results.

Our pre-registered predictions (https://osf.io/7vqar) were that: greater government restrictions in response to COVID-19, including those on social interactions (H1), and lower age (H2) would lead to greater increases in mental health symptoms across time (i.e., T1 – T3). Additionally, we predicted that these associations between mental health problems and age and country would be partially accounted for by social connectedness, and that this relationship would be moderated by social rejection sensitivity (H3). That is, heightened mental health problems in individuals residing in a country with greater COVID-19 restrictions and those of younger age would vary as a function of decreased social connectedness. This association in turn was predicted to be strongest in individuals who experience higher levels of social rejection sensitivity.

## Method

### Participants

A total of 3,208 participants consented and were eligible to take part in the CORAL study. Participants were recruited via social media advertising, advertisements in school newsletters, registration of the study on various online research platforms (e.g., MQ participate platform, SANE Australia Forum, COVID Minds, NIHR, Children Helping Science, and Assessment of COVID-19 Experiences for Adolescents), and by word of mouth. Additionally, we emailed the study information to relevant organisations (e.g., general and youth mental health organisations, pregnancy and parenting organisations), a number of which agreed to share the study information through their platforms (e.g., in newsletters, on social media). Participants had three chances (T1, T2, T3) to win an AUD $100 (£50 OR US$60) gift voucher.

To be eligible, participants needed to: a) be residing in Australia, UK, or USA, b) be 11 years or older, c) be fluent in English, d) have no history of neurodevelopmental or neurological disorder, e) have no history of traumatic brain injury (TBI), and e) be capable of providing informed consent. For the current study, participants were excluded if they had more than 20%; missing data on the mental health outcomes at T1 (*n* = 841^1^), had additional duplicate records in the dataset (T1: *n* = 9, T2: *n* = 21, T3: *n* = 13), or responded incorrectly to more than one attention check item (T1: *n* = 8, T3: *n* = 1). The final sample for the current study included *N* = 2,367 participants (89.95%; female, *M* (*SD*)_age_ = 38.85 (16.94) years). See Table 1 for a summary of participant characteristics and Table 2 for descriptive characteristics of key study variables. See Supplementary Materials (SM) 1 for further information on participant attrition.

### Measures

Only those measures included in the analyses for the current study are reported below (see SM2 for a complete list of all measures included in the CORAL study). All self-report measures had acceptable internal consistency, which is reported below. Revell’s total omega (*ωT*), which has been shown to overcome the limitations and stringent assumptions of Cronbach’s alpha, was used to measure internal consistency (McNeish, 2018). While there is no universal guide to evaluate *ωT*, scores of 0.50 and higher are considered to reflect acceptable internal consistency (Watkins, 2017).

#### Demographics

Participants provided a series of demographic measures, including age, self-identified gender, self-identified ethnicity, and highest educational attainment, which was used as a proxy for socio-economic status. For participants under the age of 18, the average of their parent’s/guardian’s highest educational attainment was used as a proxy for socio-economic status.

#### Government Restrictions in Response to COVID-19

**Figure 1:**
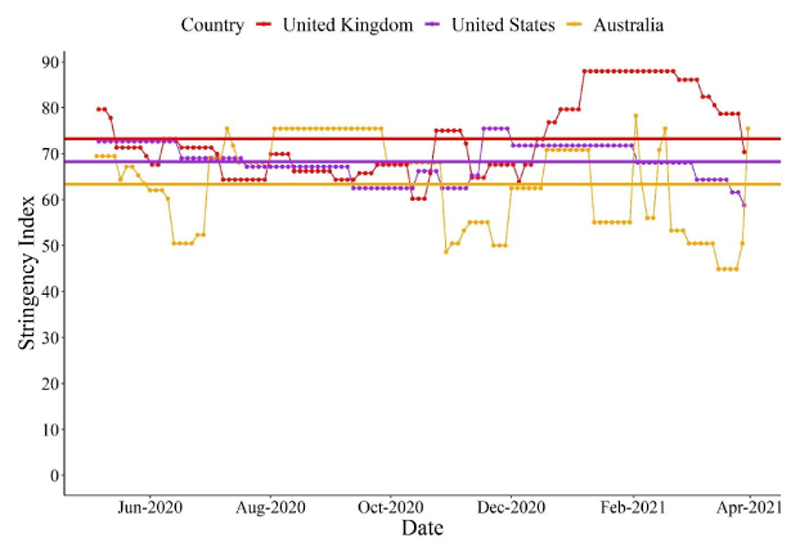
Government Stringency in Response to COVID-19 in the United Kingdom, United States of America, and Australia *Note.* The Stringency Index is a composite measure of nine indices (i.e., school closures, workplace closures, cancellation of public events, restrictions on gatherings, closure of public transport, stay at home requirements, restrictions on internal movements, international travel controls, and the presence of public information campaigns) computed by the Oxford Coronavirus Government Response Tracker project (Hale et al., 2021; for details see https://ourworldindata.org/covid-government-stringency-index) that indexes the magnitude of government response to COVID-19. The horizontal lines on the figure represent the mean stringency index in the UK, USA, and Australia between 5^th^ May 2020 and 31^st^ March 2021.

Participants’ country of residence (Australia, UK, or USA) was used as a proxy for government restrictions in response to COVID-19 (i.e., stringency index; Hale et al., 2021). The stringency index is a composite measure of nine indices (i.e., school closures, workplace closures, cancellation of public events, restrictions on gatherings, closure of public transport, stay at home requirements, restrictions on internal movements, international travel controls, and the presence of public information campaigns) computed by the Oxford Coronavirus Government Response Tracker project (Hale et al., 2021), a database of international policy responses to COVID-19 (for details see https://ourworldindata.org/covid-government-stringency-index). From the study commencement date until the date of study pre-registration, the average stringency index in the UK was 73.3; in the USA, 68.5; and in Australia, 63.6 (on a scale of 0 -100, with 100 reflecting the greatest stringency; Figure 1). Given the approximately equidistant values between countries, in the current study stringency was rank ordered from 3 (highest stringency) to 1 (lowest stringency). In addition, participants were asked the extent to which they were adhering to physical distancing measures in place in their community, rated on a scale from 1 (*Not at all*) to 6 (*Extremely*), which was controlled for in analyses.

#### COVID-19 Risk

Binary response items (0 = no, 1 = yes) assessing whether participants or anyone in their home had been quarantined due to possibly having COVID-19; whether participants had been hospitalized due to COVID-19; or whether participants knew anyone personally who had been diagnosed with, hospitalized due to or passed from COVID-19 were included in the study. These items were combined into a COVID-19-risk variable, to control for the potential impact of COVID-19-related risk on mental health.

#### Social Rejection Sensitivity

The Online and Offline Social Sensitivity Scale (O^2^S^3^; Andrews et al., 2021) was used as a measure of social rejection sensitivity. The 18-item scale assesses social rejection sensitivity in both off- and on-line contexts. Respondents indicated the extent to which they endorse such items as “I always expect criticism” on a 4-point Likert scale, ranging from 0 (*Strongly disagree*) to 3 (*Strongly agree*). The scale has shown good internal consistency (*ωT* = 0.90 – 0.93) and good convergent validity with symptoms of emotional disorders (*r* = 0.46 – 0.41; Andrews et al., 2021). The O^2^S^3^ demonstrated good internal consistency in the current study (*ωT* = 0.93). This scale was only administered at T1.

#### Social Connectedness

Social connectedness throughout the COVID-19 pandemic was assessed using several metrics including the UCLA Loneliness Scale and a series of bespoke items indexing change in face-to-face and technology-assisted interactions as well as social support from family and friends during the pandemic.

The UCLA Loneliness Scale (Russell et al., 1978) is a 20-item self-report measure designed to assess subjective feelings of loneliness as well as feelings of social isolation. Participants responded to such items as, “I am unhappy doing so many things alone” on a 4- point Likert scale, ranging from 0 (*I never feel this way*) to 3 (*I often feel this way*). The scale has demonstrated good psychometric properties, including high internal consistency and test-retest reliability (Russell et al., 1978). The scale had good internal consistency in the current sample (*ωT* = 0.97 at all timepoints). This scale was administered at all timepoints.

The bespoke items asked participants to indicate on a visual analogue scale ranging from 0 (*Much decreased*) – 100 (*Much increased*) (recoded to 1 – 5 for the current study) the extent to which the following had changed since the start of the pandemic (for T1) or since the previous time point (for T2 and T3): face-to-face contact with friends; playing video games; messaging or texting on a mobile phone, tablet, or computer; visiting social media sites; video calling/chatting; voice calling/chatting; getting support from friends; getting support from family members. The interaction items showed acceptable internal consistency at each time point (*ωT* = 0.75 – 0.95)^2^.

#### Mental Health Problems

Symptoms of depression were assessed with the 8-item Patient Health Questionnaire (PHQ-8; Kroenke et al., 2001). The measure has been shown to be a reliable index of depression and has demonstrated excellent psychometric properties (Beard et al., 2016; Martin et al., 2006). Participants indicated the extent to which they had been bothered by such things as “Little interest or pleasure in doing things” in the previous two weeks on a 4-point Likert scale, ranging from 0 (*Not at all*) to 3 (*Nearly every day*). The measure demonstrated good internal consistency in the current study (*ωT* = 0.93 – 0.94).

Additionally, the 7-item General Anxiety Disorder scale (GAD-7; Spitzer et al., 2006) was administered to assess symptoms of anxiety. Participants were asked to indicate how often they had been bothered by such problems as “Feeling nervous, anxious, or on edge” over the previous two weeks on a 4-point Likert scale, ranging from 0 (*Not at all*) to 3 (*Nearly every day*). The GAD-7 has evidenced good reliability and validity (Beard & Bjorgvinsson, 2014; Lowe et al., 2008), and good internal reliability was observed in the current study (*ωT* = 0.95 – 0.96).

Finally, mental well-being was assessed with the 7-item Short Warwick-Edinburgh Mental Wellbeing Scale (WEMWBS; Stewart-Brown et al., 2009), which has shown good psychometric properties from adolescence to older age (Stewart-Brown et al., 2009). This measure was reverse scored for analyses. Good internal reliability was observed in the current study (*ωT* = 0.89 – 0.91). Mental health measures were administered at all timepoints.

### Procedure

This study was approved by the University of New South Wales Human Research Ethics Committee (HC200287). Prior to taking part in the study, all participants were required to provide online informed consent. Participants under the age of 18 years required parental consent before they were able to access the study. Parents completed an online consent form and were then provided with a link and study access code for their child. Participants then completed the online survey on Qualtrics (Qualtrics, Provo, UT) and cognitive task (not reported here) on the Cognitron platform. Participants first provided demographic information, after which they completed the social interaction and social support items, followed by the UCLA Loneliness Scale, the PHQ-8, the GAD-7, the WEMWBS, and finally the O^2^S^3^.

### Data Analysis

Prior to hypothesis testing, preliminary analyses were conducted to model mental health problems and social connectedness as latent variables (SM 3) and to subsequently test for measurement invariance of these latent variables (SM 4). Our pre-registered method was to fit one mental health latent variable, with items from the PHQ-8, GAD-7, and WEMWBS loading on to a single unidimensional factor, and a separate social connectedness latent variable, with items from the UCLA Loneliness Scale and the social interactions and social support items loading on to a unidimensional factor. While a single mental health latent variable fit the data (SM3), a unidimensional social connectedness latent variable did not fit the data due to low correlations between items from different measures (i.e., covariance between loneliness items and interactions items = 0.002, *p* = .077; SM3). As such, the loneliness items, interactions items and social support items were modelled as separate latent variables. All latent variables demonstrated measurement variance across countries (SM4). Predicted values for each latent variable were extracted and used in all analyses (SM3).

Trajectories of change in mental health problems were analysed using latent growth curve modelling (Kievit et al., 2018). To address our first hypothesis, that individuals residing in countries with greater government stringency in response to COVID-19 would show greater increases in mental health problems across time (i.e., T1 to T3), a multi-group latent growth curve model was specified, with country of residence specified as the categorical grouping factor. The intercept was coded as 1 for each timepoint, and slope was coded linearly as 0, 1, 2. The intercept of the model thus represents mental health problems at T1, whereas the slope represents linear change across time (i.e., T1 to T3). Mental health problems were modelled as a latent factor score, with depression symptoms, anxiety symptoms, and well-being items (reverse coded) loaded onto individual factors, followed by the three factors loaded onto an overall mental health general factor (SM1). Higher scores on this latent factor score thus indicate greater mental health problems. To determine whether individuals from countries with differing levels of government stringency demonstrated distinct mental health trajectories, a model comparison approach was adopted. First, three separate multi-group models were fit: one which allowed the intercept to differ across countries (freed intercept model), one which allowed the slope to differ across countries (freed slope model), and one which constrained both the intercept and slope to be the same across countries (constrained model). The fit statistics for the freed intercept and slope models were then compared to the fit statistics of the constrained model. Significant differences between countries in baseline mental health and rate of change in mental health across time were indicated by a significant chi-squared test between the freed intercept and constrained model and between the freed slope and constrained model, respectively. In all models, gender (dummy coded as female = 1, other = 0), ethnicity (dummy coded as White = 1, other = 0), COVID-19 risk at T1, and adherence to physical distancing at T1 were controlled for^3^. The impact of these covariates was constrained across countries, as were the error variances for the intercept, slope, and mental health latent factors at each time point.

To address our second hypothesis, that younger individuals would show greater increases in mental health problems across time, age was added as a continuous time-invariant covariate (constrained across countries) to the best-fitting multi-group model, predicting both intercept and slope. In this model, predictors of intercepts address interindividual differences in mental health problems at T1, whereas predictors of slope address intraindividual differences in changes in mental health problems across time.

To address our third hypothesis, that the impact of age and country on mental health problems would be mediated by social connectedness, a series of longitudinal mediation models were specified, including age or country (numeric variable rank ordered as described in Measures section) as the predictor and mental health problems at T3 as the outcome variable. Change in loneliness, interactions and social support from T1 to T3 was included as the mediator (in separate models). Thus, a total of six mediation models were specified. All models controlled for mental health problems at T1, as well as gender, ethnicity, T1 COVID-19 risk and T1 adherence to physical distancing. A moderated mediation was planned as a second step, including social rejection sensitivity as the moderator, if any significant indirect effects were observed. This deviates from our pre-registered method of analysis, which had proposed a moderated mediation latent growth curve model. However, due to the added number of latent factors the moderated mediation model became too complex to interpret, and longitudinal mediation was instead adopted.

All analyses were conducted in R Studio version 4.1.0 using the *lavaan* package (Rosseel, 2012) and the *interactions* package (Long, 2021), using robust full information maximum likelihood estimation to account for missing data. Model fit was assessed using standard criteria, with acceptable fit indicated by comparative fit index (CFI) and Tucker-Lewis index (TLI) values ≥ .90, and root mean square error of approximation (RMSEA) and standardized root mean square residual (SRMR) values ≤.08 (Schermelleh-Engel et al., 2003). Figures were made using ggplot2 (Wickham, 2016) and ggpubr (Kassambara, 2020). An R script containing the analyses code can be found on the Open Science Framework (https://osf.io/d5yxp/).

## Results

### Hypothesis 1: The Impact of Government Restrictions on Mental Health Problems Across One Year of the Pandemic

To address H1, that changes in mental health problems across the pandemic would vary as a function of country of residence (as a proxy of government restrictions in response to the pandemic), a multi-group latent growth curve model was specified, controlling for gender, ethnicity, COVID-19 risk, and physical distancing adherence. COVID-19 risk, measured as a composite score of exposure, hospitalisation and COVID-19 mortality in next of kin and friends, was included in the model to control for impact of existential threat on mental health problems. Adherence to physical distancing restrictions was included as a covariate in the model, to model interindividual variance in adherence across countries.

A model with linear slope in which the intercept of mental health problems was allowed to vary across countries (freed intercept model) fit significantly better than a model in which the intercept was constrained across countries (*Χ*^2^_diff_ = 37.52, *df_diff_* = 2, *p* < 0.001), indicating that participants residing in the UK, the USA and Australia showed differing levels of mental health problems across the first months of the pandemic (Table S2). Specifically, individuals in the UK showed the greatest level of mental health problems (*B* = 0.16), followed by individuals in the USA (*B* = 0.07) and Australia (*B* = −0.08).

**Figure 2:**
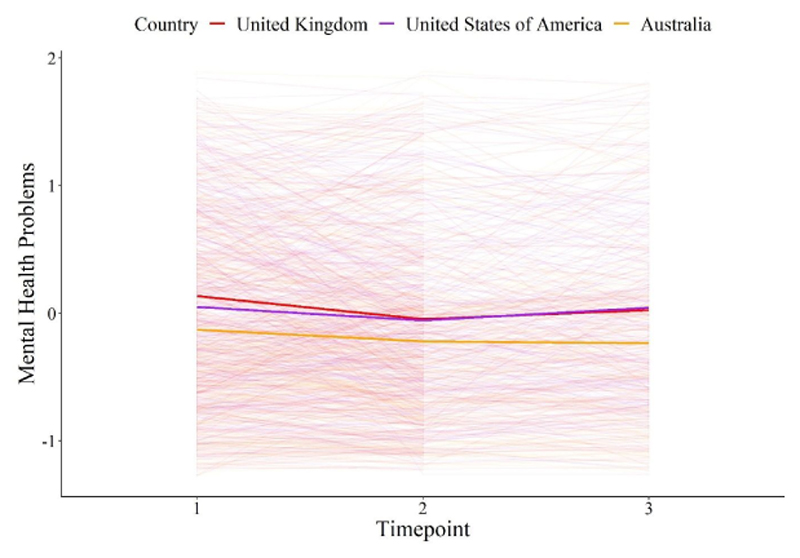
Change in Mental Health Problems as a Function of Country of Residence *Note.* Figure 2 depicts mental health problems across the first year of the pandemic from May 2020 to April 2021. The solid lines represent country averages across Australia in yellow, the UK in red and the USA in purple. The thin lines represent the mental health of individual participants. Mental health was a latent factor score comprising depression symptoms, measured with the 8-item Patient Health Questionnaire (Kroenke et al., 2001), anxiety symptoms, measured with the 7-item General Anxiety Disorder Scale (Spitzer et al., 2006), and mental wellbeing, measured with the 7-item Warwick Edinburgh Mental Wellbeing Scale (Stewart-Brown et al., 2009). Time 1 was between May 5^th^ 2020 and September 30^th^ 2020, Time 2 was between August 5^th^ 2020 and January 29^th^ 2021 and Time 3 was between November 5^th^ 2020 and April 9^th^ 2021. The countries varied in government-imposed COVID-19 restrictions, with the UK reporting the highest level of government restrictions, the USA intermediate levels and Australia reporting the lowest levels of government restrictions during the study period.

A model in which the slope of mental health problems was allowed to vary across countries (freed slope model) did not fit significantly better than a model in which the slope was constrained across countries (*Χ*^2^_diff_ = 4.65, *df_diff_* = 2, *p* = 0.098). Thus, participants residing in countries with differing levels of government restrictions in response to COVID-19 did not show differential *changes* in mental health problems across one year of the pandemic (Figure 2). Indeed, the slope was not significant (*B* = −0.00), indicating that there was no significant change in mental health problems across the course of the study. The freed intercept model demonstrated an excellent fit to the data (*Χ*^2^ = 69.79, *p* = 0.006, CFI = 0.98, TLI = 0.98, RMSEA = 0.03, SRMR = 0.03).

### Hypothesis 2: The Impact of Age on Mental Health Problems Across One Year of the Pandemic

To address H2, that changes in mental health problems across the pandemic would vary as a function of age, age was added as a time-invariant predictor (constrained across countries) to the best-fitting multi-group model (i.e., the freed intercept model). The model provided an excellent fit to the data (*Χ*^2^ = 75.67, *p* = 0.011, CFI = 0.99, TLI = 0.98, RMSEA = 0.03, SRMR = 0.03). Again, the slope was not significant (*B* = 0.00; Table 1), indicating that, overall, mental health problems remained stable from T1 to T3. Age significantly predicted the intercept (*B* = −0.01) but not the slope (*B* = −0.00; Table 3). That is, younger individuals experienced significantly greater mental health problems in the early stages of the pandemic, compared to older individuals, with these inequalities in mental health problems remaining constant across one year of the pandemic. At T1, adolescents (individuals aged 24 years and younger; Sawyer et al., 2018) reported mental health symptoms that place them in the moderate clinical range for both depression (*M* = 13.29, *SD* = 5.96) and generalized anxiety disorder (*M* = 10.97, *SD* = 6.05) based on pre-pandemic norms (Kroenke & Spitzer, 2002; Spitzer et al., 2006) (see Table S3 for scores at T2 and T3). Adults (25 years and over) scored in the mild clinical range (depression: *M* = 8.47, *SD* = 6.05, generalized anxiety: *M* = 7.50, *SD* = 6.02).

### Hypothesis 3: The Relationship Between Country, Age, Social Connectedness, and Mental Health Problems

Next, a series of longitudinal mediation models were specified to address H3, that the impact of age and country on mental health problems would be partially accounted for by social connectedness and would vary as a function of social rejection sensitivity. We first investigated the mediating effect of the social connectedness variables on the impact of age and country on mental health problems.

#### Country, Social Connectedness, and Mental Health

Neither changes in loneliness (standardised indirect effect: *B* = 0.02, *SE* = 0.02, *z* = 1.35, *p* = 0.177; Figure S1), frequency of social interactions (standardised indirect effect: *B* = 0.00, *SE* = 0.002, *z* = 0.05, *p* = 0.959; Figure S2), nor social support (standardised indirect effect: *B* = 0.004, *SE* = 0.004, *z* = 1.31, *p* = 0.189; Figure S3) mediated the relationship between country and mental health problems at T3, while controlling for mental health problems at T1, gender, ethnicity, T1 COVID-19 risk, and T1 adherence to physical distancing measures. In each model, there was no significant direct effect of country on mental health problems at T3 (loneliness model: standardised *B* = −0.01, *SE* = 0.03, *z* = −0.30, *p* = 0.766; interactions model: standardised *B* = 0.02, *SE* = 0.03, *z* = 0.90, *p* = 0.367; social support model: standardised *B* = 0.02, *SE* = 0.03, *z* = 0.71, *p* = 0.475). There was also no effect of country on changes in loneliness (standardised *B* = 0.05, *SE* = 0.04, *z* = 1.34, *p* = 0.181), frequency of social interactions (standardised *B* = 0.002, *SE* = 0.05, *z* = 0.05, *p* = 0.959), or social support (standardised *B* = −0.05, *SE* = 0.05, *z* = −1.42, *p* = 0.156). Similarly, there was no effect of changes in frequency of social interactions on mental health problems at T3 (standardised *B* = 0.04, *SE* = 0.02, *z* = 1.49, *p* = 0.136), suggesting that whether or not individuals increased or decreased their face-to-face and virtual interactions with others during the pandemic did not significantly impact on mental health problems at T3. However, there was a significant, moderate effect of changes in loneliness (standardised *B* = 0.35, *SE* = 0.03, *z* = 13.54, *p* < 0.001) and a significant, small effect of changes in social support (standardised *B* = −0.08, *SE* = 0.03, *z* = −2.81, *p* = 0.005) on mental health problems at T3. Thus, greater increases in loneliness across time predicted greater mental health problems after one year of the pandemic, whereas greater increases in social support predicted fewer mental health problems after one year of the pandemic.

#### Age, Social Connectedness, and Mental Health

When examining the indirect effect of social connectedness on the relationship between age and mental health problems at T3, no significant indirect effects were observed (loneliness standardised indirect effect: *B* = −0.01, *SE* = 0.001, *z* = −0.90, *p* = 0.368, Figure S4; frequency of social interactions standardised indirect effect: *B* = 0.004, *SE* = 0.00, *z* = 1.55, *p* = 0.122, Figure S5; social support standardised indirect effect: *B* = 0.001, *SE* = 0.00, *z* = 0.57, *p* = 0.569, Figure S6). However, in each model there was a significant, small effect of age on mental health problems at T3 (loneliness model: standardised *B* = −0.06, *SE* = 0.001, *z* = −3.26, *p* = 0.001; interactions model: standardised *B* = −0.10, *SE* = 0.001, *z* = −4.64, *p* < 0.001; social support model: standardised *B* = −0.10, *SE* = 0.001, *z* = −4.52, *p* < 0.001). Thus, younger age predicted significantly greater mental health problems at T3, over and above mental health problems at T1, gender, ethnicity, and COVID-19 risk and physical distancing adherence at T1. Age also significantly predicted changes in the frequency of social interactions (standardised *B* = 0.09, *SE* = 0.002, *z* = 2.67, *p* = 0.008), but not change in loneliness (standardised *B* = −0.03, *SE* = 0.002, *z* = −0.90, *p* = 0.370) or social support (standardised *B* = −0.02, *SE* = 0.002, *z* = −0.57, *p* = 0.566). As in the country models, there was a moderate effect of changes in loneliness (*B* = 0.35, *SE* = 0.03, *z* = 13.08, *p* < 0.001), a small effect of social support (*B* = −0.07, *SE* = 0.03, *z* = −2.77, *p* = 0.006) and a non-significant effect of frequency of social interactions (*B* = 0.05, *SE* = 0.02, *z* = 1.91, *p* = 0.056) on mental health problems at T3.

#### The Moderating Role of Social Rejection Sensitivity

Across the mediation models, younger age significantly predicted more mental health problems, as did changes in loneliness and social support. We next investigated the moderating role of social rejection sensitivity on each of these relationships. The effects of loneliness and social support were investigated in separate linear models and a model comparison approach was adopted to determine the significance of interaction effects. This differs from our pre-registered method of analysis, which had proposed a moderated (social rejection sensitivity as moderator) mediation (changes in social connectedness as mediator) latent growth curve model. However, given that we did not find significant mediation effects of social connectedness on the association between age and country with mental health, we instead investigated the impact of the moderator (i.e., social rejection sensitivity) through linear models.

**Figure 3:**
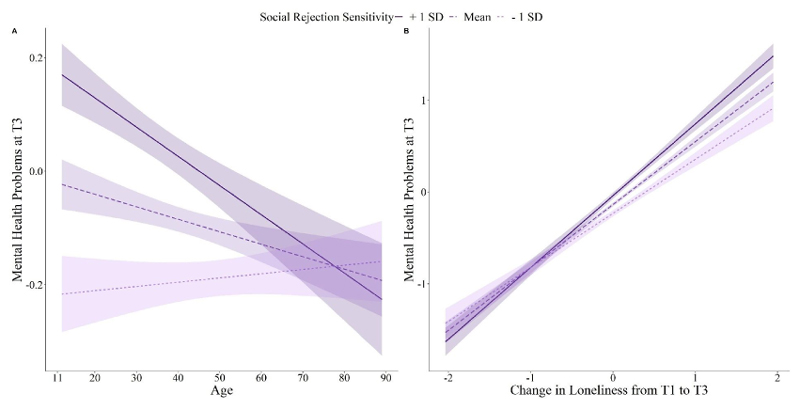
Effect of Age and Change in Loneliness on Mental Health Problems as a Function of Social Rejection Sensitivity *Note.* Figure 3 depicts the moderating impact of social rejection sensitivity on the association between age (3A) and change in loneliness (3B) with mental health at T3 (January 2021 – April 2021) controlling for mental health problems, COVID-19 risk, and physical distancing adherence at T1 (May 2020-September 2020), country, gender, and ethnicity. Mental health problems were modelled as a latent factor score comprising depression symptoms, measured with the 8-item Patient Health Questionnaire (Kroenke et al., 2001); anxiety symptoms, measured with the 7-item General Anxiety Disorder Scale (Spitzer et al., 2006); and mental wellbeing, measured with the 7-item Warwick Edinburgh Mental Wellbeing Scale (Stewart-Brown et al., 2009). Social rejection sensitivity was measured with the 18-item Online and Offline Social Sensitivity Scale (Andrews et al., 2021). Loneliness was measured with the 20-item UCLA Loneliness Scale (Russell et al., 1978) and modelled as a latent factor score.

First, a main effects model, in which age, loneliness, and social rejection sensitivity predicted mental health at T3, controlling for mental health problems, COVID-19 risk, and physical distancing adherence at T1, country, gender, and ethnicity, was compared to a model which additionally included an interaction between social rejection sensitivity and loneliness. The addition of the interaction term significantly improved model fit (*F* = 6.61, *p* = 0.010). The main effects model was also compared to a model which included an interaction between social rejection sensitivity and age. Again, the addition of the interaction term significantly improved model fit (*F* = 11.11, *p* < 0.001). A final linear model was specified, including the aforementioned main effects and covariates, as well as the two interaction terms. In this model, age (*B* = 0.004, *SE* = 0.002, *t* = 2.07, *p* = 0.039), loneliness change (*B* = 0.5, *SE* = 0.10, *t* = 5.05, *p* < 0.001) and social rejection sensitivity (*B* = 0.02, *SE* = 0.004, *t* = 5.25, *p* < 0.001) significantly predicted mental health problems at T3. These main effects were qualified by a significant interaction between social rejection sensitivity and age (*B* < −0.001, *SE* < 0.001, *t* = −3.35, *p* < 0.001; Figure 3A) and between social rejection sensitivity and loneliness (*B* = 0.01, *SE* = 0.004, *t* = 2.59, *p* = 0.010; Figure 3B).

Simple slopes analyses revealed that age had a significant effect on mental health problems when social rejection sensitivity was average (*B* = – 0.002, *SE* = 0.001, *t* = −2.21, *p* = 0.027) or high (+1 SD; *B* = −0.005, *SE* = 0.001, *t* = −3.67, *p* < 0.001), but not when social rejection sensitivity was low (–1 SD; *B* = 0.001, *SE* = 0.001, *t* = 0.60, *p* = 0.549). The effect of change in loneliness on mental health problems was significant across all levels of social rejection sensitivity; however, this effect was greater the higher the social rejection sensitivity (low SRS: *B* = 0.59, *SE* = 0.06, *t* = 10.29, *p* < 0.001; average SRS: *B* = 0.69, *SE* = 0.04, *t* = 16.42, *p* < 0.001; high SRS: *B* = 0.78, *SE* = 0.06, *t* = 14.28, *p* < 0.001).

To investigate the effect of change in social support on mental health problems, a main effects model, including age, social support, and social rejection sensitivity as predictors, and controlling for mental health problems, COVID-19 risk, and physical distancing adherence at T1, country, gender, and ethnicity, was compared to a model which included an interaction between social rejection sensitivity and social support. The inclusion of the interaction term did not significantly improve model fit (*F* = 1.18, *p* = 0.278). When comparing the main effects model to a model which included an interaction between social rejection sensitivity and age, the inclusion of the interaction term significantly improved model fit (*F* = 4.55, *p* = 0.033). Thus, the linear model including the aforementioned main effects, covariates, and an interaction between social rejection sensitivity and age was retained. In this model change in social support (*B* = −0.09, *SE* = 0.03, *t* = −2.74, *p* = 0.006) and social rejection sensitivity (*B* = 0.02, *SE* = 0.005, *t* = 4.18, *p* < 0.001) significantly predicted mental health problems at T3, over and above the effect of mental health problems, COVID risk, and physical distancing adherence at T1, country, gender, and ethnicity. The main effect of age was not significant (*B* = 0.001, *SE* = 0.003, *t* = 0.44, *p* = 0.663), however, again there was a significant interaction between social rejection sensitivity and age (*B* < – 0.001, *SE* < 0.001, *t* = −2.13, *p* = 0.033).

## Discussion

Brain, social and cognitive development is driven by our experiences (Holtmaat & Svoboda, 2009). During adolescence, peer interactions are central to experience-dependent neural and socioemotional development (Blakemore, 2018). This developmental process was disrupted by the advent and ongoing nature of the COVID-19 pandemic. The detrimental impact of enforced social isolation on young people’s mental health needs to be dissected. Here we examined the impact of known social risk and protective factors prospectively. As predicted, levels of government-enforced COVID-19 restrictions and age were associated with mental health problems. Specifically, individuals living in a country with higher government stringency reported worse mental health problems throughout the study period, even when controlling for COVID-19-risk and adherence to physical distancing regulations. Younger age was also associated with more mental health problems at each of the three assessment timepoints. Increased loneliness across the three study assessment points was associated with more mental health problems at the final time point, whereas increased social support was associated with fewer mental health problems. Changes in frequency of face-to-face and online social interactions were not significantly related to mental health outcomes in the current study. The impact of age and loneliness on mental health problems was moderated by social rejection sensitivity. Younger age in individuals with moderate to high levels of social rejection sensitivity was associated with more mental health problems at the final assessment, but not in those with low levels of social rejection sensitivity. Increases in loneliness across the study period were associated with the worst mental health outcomes in those individuals highest in social rejection sensitivity. Together these findings demonstrate that social risk and protective factors are key determinants of mental health during the COVID-19 pandemic, especially in young people.

Since the emergence of COVID-19 and subsequent enforcement of strict and extended lockdown measures to combat the virus, mental health researchers have warned of an impending mental health crisis (Holmes et al., 2020), what has now been termed a ‘shadow pandemic’ (Editorial, 2021). Here we found that individuals residing in countries with greater government stringency in response to the pandemic, including those in the UK and USA, demonstrated consistently higher mental health problems across the first year of the pandemic. Moreover, in the current study we observed substantially heightened depression and anxiety symptoms at all time-points, when compared to pre-pandemic norms (Kroenke et al., 2001; Spitzer et al., 2006). Indeed, average depression scores in adults at the first assessment time point were in the mild clinical range and in the upper moderate clinical range for adolescents. Even when considering self-selection to participate in a psychological study on the impact of COVID-19 on mental well-being, these are exceptionally high average levels of depression. While the observational nature of the current study and lack of prepandemic data and direct measurement of physical distancing precludes causal interpretations, together these findings do suggest that the COVID-19 pandemic and ensuing lockdowns have had a significant impact on mental health, a pattern observed in a growing number of longitudinal studies including pre-pandemic data (Barendse et al., 2021; Chahal et al., 2020; De France et al., 2021; Magson et al., 2020; Pierce, Hope, et al., 2020), especially in young people (Racine et al., 2021).

In line with our pre-registered predictions and a growing body of research (Cooper et al., 2003; Groarke et al., 2021; Hoffart et al., 2020; Hyland et al., 2021; Killgore, Cloonan, Taylor, Miller, et al., 2020; Magson et al., 2020; Palgi et al., 2020; Rodman et al., 2021) mental health problems, specifically increased symptoms of depression and generalized anxiety and reduced well-being, were associated with increases in loneliness throughout the pandemic. While we did not investigate the reciprocal nature of this relationship, recent research conducted during the pandemic found a bidirectional relationship between loneliness and depressive symptoms (Groarke et al., 2021). Indeed, a recent qualitative synthesis of studies of the experience of loneliness among young people with depression highlighted the mutually reinforcing nature of this relationship (Achterbergh et al., 2020). The authors argue that the manner in which depressed individuals engage in certain behaviours, such as withdrawing and not confiding in others about their symptoms, can lead to feelings of loneliness. An awareness of these feelings of loneliness can in turn perpetuate depressed individuals’ negative mood states. Thus, mental health interventions that target loneliness may lead to improvements in symptoms, and vice versa.

Our results extend this general finding by investigating and providing support for the hypothesis that loneliness is particularly detrimental to those characterized by heightened levels of social rejection sensitivity. That is, increased loneliness impacted those participants most who were most sensitive to social rejection. This fits with theoretical models of social rejection sensitivity, which argue that heightened social rejection sensitivity is marked by excessive distress in response to social uncertainty and ambiguity (Allen & Badcock, 2003; Downey & Feldman, 1996). Ever changing social distancing directions, including school closures and working from home orders, have introduced high levels of uncertainty regarding the potential for social contact. Indeed, isolation is likely to be interpreted by rejection sensitive individuals as a rejection signal (Molinari et al., 2020).

As a group, adolescents feel social rejection particularly keenly (Sebastian et al., 2010). The current results showed that the association between mental health across one year of the pandemic and age was moderated by social rejection sensitivity. That is, young people high on social rejection sensitivity showed the highest levels of mental health problems after one year of the pandemic, whereas in those with low levels of social rejection sensitivity, age was not significantly related to mental health outcomes. Again, this supports the proposal that the detrimental impact of social isolation and uncertainty experienced within the context of the current pandemic was greatest in those high in social rejection sensitivity. The current findings thus suggest that social rejection sensitivity may be an important intervention target, especially for young people. While little research has investigated the malleability of social rejection sensitivity, there is preliminary evidence that negative interpretation bias mediates the association between the tendency to anxiously anticipate rejection and depressive symptoms (Normansell & Wisco, 2017). Importantly, meta-analytic evidence suggests that negative interpretation bias can be modified via targeted training (Menne-Lothmann et al., 2014). Thus, interventions that challenge negative interpretations about social rejection may help decrease mental health symptoms in those sensitive to rejection.

In the absence of opportunities for face-to-face contact during periods of physical distancing, many individuals may have turned to virtual means, such as social media, in order to maintain a sense of social connectedness. However, whether such virtual platforms help or hinder mental health has been a source of much debate in the literature (Naslund et al., 2020). Our findings suggest that whether or not individuals increased or decreased their face-to-face and virtual interactions with others during the pandemic did not significantly impact on their mental health. This is consistent with some recent findings showing that the *duration* or *quantity* of interactions with others on virtual platforms is not associated with mental health outcomes during the pandemic (Bernasco et al., 2021; K. Cooper et al., 2021; Rodman et al., 2021; Rosenberg et al., 2021; Towner et al., 2021). Instead, it may be the *quality* of virtual interactions that is important (Magis-Weinberg et al., 2021). More generally, increased social support was associated with fewer mental health problems in the current study, which is in line with previous findings during the pandemic (Bernasco et al., 2021; Iob et al., 2020; Rodman et al., 2021).

These findings need to be interpreted within the context of the study’s limitations. First, we collected a convenience, non-probability sample and consequently our findings may not be representative of the population. Indeed, with the rapid proliferation of research on the mental health impact of COVID-19, concerns have arisen as to whether non-probability samples provide an accurate representation of a population response (Pierce, McManus, et al., 2020). Consequently, our findings may be biased as a consequence of the people who are likely to take part in such surveys; for example, those who are more engaged and interested in the topic and also have access to the internet. Moreover, most participants identified as female, White and were of mid-high socioeconomic status, limiting the generalizability of these findings. Female gender in particular could be argued to be associated with greater social rejection sensitivity. However, meta-analytic evidence provides no support for gender differences in the association between social rejection sensitivity and mental health (i.e., depression symptoms; Gao et al., 2017). Similarly, a previous study in a sample of participants with high ethnic, socioeconomic and gender diversity showed that the impact of these demographic variables did not moderate the impact of social rejection sensitivity on mental health (Minihan et al., 2021). While we included self-identified gender and ethnicity as covariates in all analyses, the lack of representativeness of our sample (i.e., majority White and female) limits the conclusions we can draw about the impact of these demographic variables on mental health trajectories.

Another limitation of the current study was that we did not include a direct measure of physical distancing at each timepoint. Instead, the stringency index (Hale et al., 2021; described in Methods) was used as an overall indicator of country-level physical distancing. Future studies should model time-variant social restrictions combined with more finely differentiated geo-localization to support the current findings. Additionally, our study did not include pre-pandemic data, which limits the conclusions that can be drawn from our findings regarding the direct impact of the pandemic and associated restrictions. Indeed, while we found that participants residing in countries with greater government stringency reported higher mental health problems at baseline, trajectories of change in mental health problems across the course of the study did not vary as a function of government stringency. Thus, it is possible that the differences in the intercepts captured pre-existing differences in mental health problems between countries. Moreover, while we found that younger age was associated with more mental health problems at all timepoints, in the absence of pre-pandemic data, we cannot conclude that these discrepancies are a consequence of the pandemic, and do not merely reflect age-related differences in mental health. Finally, the moderating impact of social rejection sensitivity on the relationship between age and loneliness with mental health problems may not be specific to the pandemic, and instead may reflect the fact that those participants with higher social rejection sensitivity already had higher mental health problems.

Acknowledging the above limitations, the current findings add to the growing literature on patterns of mental health problems during the COVID-19 pandemic. Our findings converge with recent studies highlighting young people as being particularly vulnerable to increased mental health problems during the pandemic, and additionally highlight loneliness and social support as key risk and protective factors, respectively. Social rejection sensitivity was also found to predict increased mental health difficulties, particularly in younger people and those who experienced a greater increase in loneliness. However, despite its role as a key risk factor for adolescent-onset internalizing symptoms (Chango et al., 2012; Marston et al., 2010; Platt et al., 2013), there is currently a surprising lack of interventions directly targeting social rejection sensitivity. Thus, our findings highlight the need to design novel, easy-to-disseminate interventions targeting social rejection sensitivity.

Reducing social rejection sensitivity, especially in young people, may lead to improved resilience in the context of perceived and actual isolation and loneliness.

## Supplementary Material

Supplementary Material

## Figures and Tables

**Table 1:** Summary of Participant Characteristics

Participant Characteristics		*n* (%;)
**Age**		
	11 – 24 years old	436 (18.42%;)
	25 – 64 years old	1555 (65.69%;)
	65 – 100 years old	351 (14.83%;)
	Missing	25 (1.06%;)
**Gender Identity**		
	Female	2129 (89.95%;)
	Male	211 (8.91%;)
	Other	21 (0.89%;)
	Prefer not to say	6 (0.25%;)
**Country**		
	United Kingdom	1075 (45.42%;)
	United States of America	699 (29.53%;)
	Australia	593 (25.05%;)
**Ethnicity**		
	White	1995 (84.32%;)
	Asian	105 (4.44%;)
	Mixed	71 (3%;)
	Hispanic	44 (1.86%;)
	African	16 (0.68%;)
	Aboriginal or Torres Strait Islander	10 (0.42%;)
	Other	95 (4.02%;)
	Prefer not to say	30 (1.27%;)
	Missing	1 (0.04%;)
**SES**		
	High	1635 (69.07%;)
	Middle	654 (27.63%;)
	Low	5 (0.21%;)
	Missing	73 (3.09%;)

*Note.* SES = Socioeconomic status. For participants over the age of 18 years, SES was operationalised as their highest educational attainment. For participants under the age of 18 years, SES was operationalised as the average of their parent’s highest educational attainment; high = university, middle = high school or professional/vocational training, low = primary school.

**Table 2:** Mental Health, Loneliness, Social Interactions, Social Support

	T1 *n; M (SD*)	T2 *n; M (SD*)	T3 *n; M (SD*)
**Clinical characteristics**			
Depressive symptoms	2,366; 9.35 (6.32)	825; 8.04 (6.27)	743; 8.42 (6.32)
Anxiety symptoms	2,352; 8.14 (6.18)	822; 6.73 (5.83)	743; 7.31 (6.00)
Mental well-being	2,339; 19.64 (3.96)	819; 20.25 (4.06)	739; 19.88 (4.01)
Loneliness	2,357; 25.65 (15.75)	834; 25.66 (16.01)	753; 27.49 (15.87)
**Social interactions**			
Face-to-face interactions	2,089; 1.35 (0.66)	851; 2.24 (1.12)	744; 1.98 (1.02)
Video game interactions	1,820; 2.51 (1.35)	761; 3.26 (0.78)	700; 3.25 (0.80)
Messaging interactions	1,998; 3.02 (1.35)	814; 3.59 (0.73)	739; 3.62 (0.73)
Social media interactions	2,019; 3.20 (1.38)	816; 3.67 (0.80)	750; 3.70 (0.81)
Video calling interactions	2,024; 3.08 (1.48)	822; 3.50 (0.92)	731; 3.54 (0.95)
Voice calling interactions	1,881; 2.86 (1.41)	795; 3.38 (0.84)	716; 3.35 (0.85)
**Social support**			
Social support from friends	1,706; 2.75 (1.15)	801; 2.95 (0.85)	721; 2.86 (0.87)
Social support from family	1,764; 3.11 (1.13)	811; 3.10 (0.85)	724; 3.05 (0.87)

*Note.* Depressive symptoms measured with the 8-item Patient Health Questionnaire (Kroenke et al., 2001); Anxiety symptoms measured with the 7-item General Anxiety Disorder Scale (Spitzer et al., 2006); Mental well-being measured with the 7-item Warwick Edinburgh Mental Well-being Scale (Stewart-Brown et al., 2009); Loneliness measured with the 20-item UCLA Loneliness Scale (Russell et al., 1978); Face-to-face, video game, messaging, social media, video calling, and voice calling interactions = change in each type of interaction since the start of the pandemic (T1) or since the previous time point (T2 and T3) measured on a visual analogue scale ranging from 1 – 5; Social support from friends and family = change in social support from friends and family since the start of the pandemic (T1) or since the previous time point (T2 and T3) measured on a visual analogue scale ranging from 1 – 5.

**Table 3:** Multi-Group Latent Growth Curve Model Assessing the Impact of Age on Mental Health Problems Across One Year of the Pandemic

	*B*	*SE*	*z*	*P*
**Intercept**				
UK	0.32	0.12	2.77	0.006
USA	0.27	0.11	2.39	0.017
Australia	0.11	0.11	0.97	0.331
**Slope**	0.00	0.06	0.02	0.986
**Regressions**				
Intercept				
Age	–0.01	0.001	–12.60	< 0.001
Female	0.03	0.06	0.63	0.529
White	–0.06	0.04	–1.40	0.161
COVID-19 risk	0.03	0.02	2.02	0.043
Physical distancing adherence	0.04	0.02	2.23	0.026
Slope				
Age	–0.00	0.00	–1.17	0.244
Female	–0.04	0.03	–1.48	0.140
White	0.05	0.03	1.78	0.076
COVID-19 risk	–0.01	0.01	–1.60	0.110
Physical distancing adherence	0.00	0.01	0.03	0.974
**Variances**				
MH1	0.13	0.02	5.66	< 0.001
MH2	0.10	0.01	10.34	< 0.001
MH3	0.06	0.03	2.56	0.011
Intercept	0.37	0.02	15.57	< 0.001
Slope	0.01	0.01	1.09	0.276

*Note.* MH1 = mental health problems at T1, MH2 = mental health problems at T2, MH3 = mental health problems at T3. Mental health problems were modelled as a latent factor score comprising depression symptoms, measured with the 8-item Patient Health Questionnaire (Kroenke et al., 2001), anxiety symptoms, measured with the 7-item General Anxiety Disorder Scale (Spitzer et al., 2006), and mental wellbeing, measured with the 7-item Warwick Edinburgh Mental Wellbeing Scale (Stewart-Brown et al., 2009). COVID-19 risk was a composite score comprising a series of bespoke, binary items indexing whether participants had been quarantined or hospitalized due to COVID-19, or whether they knew anyone who had been diagnosed with, hospitalised or passed away from COVID-19. Physical distancing adherence was a bespoke item indexing the extent to which participants were complying with the physical distancing measures in place in their community, measured on a scale from 1 (*Not at all*) to 6 (*Extremely*). Due to large amounts of missing data on the COVID-19 risk and physical distancing adherence variables at T2 and T3, only T1 values of these variables were controlled for.

## Footnotes

1 The mental health measures were included in the latter half of the online questionnaire; thus, a substantial proportion of participants did not reach this point in the questionnaire and were consequently excluded from analyses.

2 Internal reliability for social support was not calculated due to this variable being assessed by only two items.

3 In line with our pre-registration, we had planned to control for level of COVID-19 risk and level of adherence to physical distancing restrictions at all timepoints. However, due to a large amount of missing data on these variables at T2 and T3, only T1 values of these variables were controlled for, allowing us to maintain our T1 sample size.
