# Supplementary material for: Social Determinants of Mental Health During a Year of the COVID-19 Pandemic
Source: Dev Psychopathol. Author manuscript; Available in PMC 2023 Nov 15. (PMC7615306; doi:10.1017/S0954579422000396)
Supplement: Supplementary Material [file EMS143989-supplement-Supplementary_Material.pdf]

# Social Determinants of Mental Health During a Year of the COVID-19 Pandemic

## Supplementary Materials

### SM 1

#### *Participant Attrition*

Of the 2,367 participants included at T1, 927 participants commenced T2 and 846 commenced T3. Participant attrition was unrelated to self-identified ethnicity and SES at both T2 and T3, as well as self-identified gender at T3; however, attrition at T2 did significantly differ as a function of self-identified gender (see Table S1).

**Table S1**

*Participant Attrition as a Function of Self-Identified Gender, Ethnicity, and Socioeconomic Status*

| | **Commenced T2** | | **Commenced T3** | | **$\chi^2$ (*p*)** | |
|---|---|---|---|---|---|---|
| | **Yes (*n*)** | **No (*n*)** | **Yes (*n*)** | **No (*n*)** | **T2** | **T3** |
| **Self-Identified Gender** | | | | | 13.40 (0.004) | 5.62 (0.132) |
| **Female** | 811 | 1318 | 747 | 1382 | | |
| **Male** | 103 | 108 | 89 | 122 | | |
| **Other** | 12 | 9 | 9 | 12 | | |
| **Prefer not to say** | 1 | 5 | 1 | 5 | | |
| **Ethnicity** | | | | | 10.57 (0.159) | 4.16 (0.761) |
| **White** | 784 | 1211 | 722 | 1273 | | |
| **Asian** | 50 | 55 | 40 | 65 | | |

| | | | | | | | |
|---|---|---|---|---|---|---|---|
| | **Mixed** | 25 | 46 | 24 | 47 | | |
| | **Hispanic** | 12 | 32 | 13 | 31 | | |
| | **African** | 8 | 8 | 7 | 9 | | |
| | **Aboriginal or Torres Strait Islander** | 3 | 7 | 3 | 7 | | |
| | **Other** | 38 | 57 | 27 | 68 | | |
| | **Prefer not to say** | 7 | 23 | 10 | 20 | | |
| **SES** | | | | | | 1.82 (0.403) | 2.42 (0.298) |
| | **High** | 655 | 980 | 607 | 1028 | | |
| | **Middle** | 247 | 407 | 223 | 431 | | |
| | **Low** | 1 | 4 | 1 | 4 | | |

**SM 2**

***Measures included in CORAL Study***

The CORAL study included a series of measures in order to assess the extent to which the COVID-19 pandemic has affected people's wellbeing, social connections, and mental abilities. Specifically, demographic characteristics including age, sex, ethnicity, education, parental education and history of mental health diagnosis were assessed. Mental health was assessed at all timepoints with the 8-item Patient Health Questionnaire, the 7-item General Anxiety Disorder Scale, and the 7-item Warwick Edinburgh Mental Well-being Scale, as described in the main manuscript. Cognitive vulnerabilities and resilience to mental health were assessed with the 12-item Intolerance of Uncertainty Scale (T1), the 18-item Online and Offline Social Sensitivity Scale (T1), the 14-item Penn State Worry scale (all timepoints), the 8-item Mental Flexibility Questionnaire (all timepoints), and the 10-item Emotion Regulation Scale (T1). COVID-19 stress exposure was assessed with a bespoke questionnaire (all

timepoints). For participants who had recently given birth, the 10-item Postpartum Distress Measure (excluding the item tapping into suicidality) and 37-item Infant Behaviour Questionnaire were administered (T2 and T3). In addition, participants were asked to complete the Affective Backward Digit Span task (all timepoints) and a social network questionnaire (all timepoints). Finally, participants who consented to use the Emotional Brain Study App, were asked to respond to four brief questionnaires assessing their mood three times daily for three days after each timepoint.

**SM 3**

***Measurement Models for Latent Variables***

A measurement model in which the PHQ-8 items, GAD-7 items and reverse-coded WEMWBS items loaded on to individual factors, with these three factors then loaded on to an overall mental health general factor, was compared to a measurement model in which the items from each scale were loaded on to a unidimensional factor. The model which differentiated the questionnaires (CFI = 0.91, TLI = 0.90, RMSEA = 0.08, SRMR = 0.05) was found to fit substantially better than the model loading all questionnaire items onto the same factor ($\chi^2_{\text{diff}} = 6604.8$, $df_{diff} = 3$; $p < 0.001$). As such, this model was retained for analyses.

Social connectedness, which was measured with the UCLA Loneliness Scale and bespoke items assessing face-to-face and online interactions with friends, as well as social support from family and friends, was unable to be modelled as a unidimensional latent variable, due to low correlations between items from different measures (i.e., covariance between loneliness general factor and interactions general factor = .002, $p = .077$). As such, the loneliness items, interactions items and social support items were modelled as separate

latent variables. All three measurement models demonstrated an adequate fit to the data (loneliness: CFI = 0.87, TLI = 0.86, RMSEA = 0.11, SRMR = 0.06; interactions: CFI = 0.95, TLI = 0.91, RMSEA = 0.14, SRMR = 0.03; social support: CFI = 1, TLI = 1, RMSEA = 0, SRMR = 0).

Finally, a complete measurement model was specified, with the PHQ-8 items, GAD-7 items, WEMWBS items, UCLA Loneliness Scale items, bespoke interactions item, and bespoke social support items loading onto separate general factors, and the PHQ-8, GAD-7 and WEMWBS general factors loading onto a mental health general factor. Predicted values for each latent variable were extracted from this model and used in all analyses.

**SM 4**

***Measurement Invariance***

Measurement invariance across countries was determined by fitting models where the loadings for the mental health and social connectedness items were constrained to be equal across countries, and comparing these models to a model where the loadings were allowed to vary across countries. Comparing the freed and constrained mental health models revealed a significant drop in model fit when constraining the measures to be equal across countries ($\chi^2_{\text{diff}}$ = 65.64, $df_{diff}$ = 42; $p$ = 0.011). The difference in model fit is not very large, and due to the large sample size even very small differences become statistically significant. A partial measurement invariance test freeing the loadings of just two WEMWBS items with the largest differences in loadings between countries supported measurement non-invariance, as the partially constrained model (in which the loadings of the items were allowed to vary across countries) did not demonstrate a deterioration in model fit when compared with the freed model ($\chi^2_{\text{diff}}$ = 52.19, $df_{diff}$ = 38; $p$ = 0.062). For subsequent analyses, to allow for the

analytical approach comparing mean differences between countries applied in the paper, the loadings for each of the mental health measures were therefore constrained to be equal across countries, and measurement invariance was not modelled in the analysis. For the social connectedness latent variable models, the constrained model did not demonstrate a deterioration in model fit when compared with the freed model ($\chi^2_{diff} = 59.74$, $df_{diff} = 48$; $p = 0.119$), indicating that the social connectedness measures were invariant across countries.

**Table S2**

*Multi-Group Latent Growth Curve Model Assessing the Impact of Country of Residence on Mental Health Problems Across One Year of the Pandemic*

| | *B* | *SE* | *z* | *p* |
|---|---|---|---|---|
| **Intercept** | | | | |
| UK | 0.16 | 0.12 | 1.34 | 0.180 |
| USA | 0.07 | 0.12 | 0.64 | 0.524 |
| Australia | −0.08 | 0.12 | −0.72 | 0.471 |
| **Slope** | −0.00 | 0.06 | −0.01 | 0.994 |
| **Regressions** | | | | |
| Intercept | | | | |
| Female | 0.05 | 0.06 | 0.95 | 0.342 |
| White | −0.16 | 0.04 | −3.67 | < 0.001 |
| COVID-19 risk | 0.03 | 0.02 | 2.10 | 0.04 |
| Physical distancing adherence | 0.003 | 0.02 | 0.18 | 0.859 |
| Slope | | | | |
| Female | −0.03 | 0.03 | −1.31 | 0.191 |

| | | | | |
|---|---|---|---|---|
| White | 0.04 | 0.03 | 1.57 | 0.118 |
| COVID-19 risk | −0.01 | 0.01 | −1.49 | 0.138 |
| Physical distancing adherence | −0.004 | 0.01 | −0.42 | 0.674 |
| **Variances** | | | | |
| MH1 | 0.13 | 0.02 | 5.95 | < 0.001 |
| MH2 | 0.10 | 0.01 | 10.17 | < 0.001 |
| MH3 | 0.07 | 0.03 | 2.73 | 0.006 |
| Intercept | 0.40 | 0.02 | 16.78 | < 0.001 |
| Slope | 0.01 | 0.01 | 0.84 | 0.401 |

*Note*. Country of residence was used as a proxy for government stringency in response to the COVID-19 pandemic. MH1 = mental health problems at T1, MH2 = mental health problems at T2, MH3 = mental health problems at T3. Mental health problems were modelled as a latent factor score comprising depression symptoms, measured with the 8-item Patient Health Questionnaire (Kroenke et al., 2001), anxiety symptoms, measured with the 7-item General Anxiety Disorder Scale (Spitzer et al., 2006), and mental wellbeing, measured with the 7-item Warwick Edinburgh Mental Wellbeing Scale (Stewart-Brown et al., 2009). COVID-19 risk was a composite score comprising a series of bespoke, binary items indexing whether participants had been quarantined or hospitalized due to COVID-19, or whether they knew anyone who had been diagnosed with, hospitalised or passed away from COVID-19. Physical distancing adherence was a bespoke item indexing the extent to which participants were complying with the physical distancing measures in place in their community, measured on a scale from 1 (*Not at all*) to 6 (*Extremely*). Due to large amounts of missing data on the COVID-19 risk and physical distancing adherence variables at T2 and T3, only T1 values of these variables were controlled for.

**Table S3**

*Symptoms of Depression and Generalized Anxiety at T2 and T3*

| | ***Adolescents*** | ***Adults*** |
|---|---|---|
| | ***M (SD)*** | ***M (SD)*** |
| **Time 2: August 2020 – January 2021** | | |
| Depression | 11.98 (6.81) | 7.27 (5.84) |
| Generalized anxiety | 9.85 (6.27) | 6.12 (5.54) |
| **Time 3: November 2021 – April 2021** | | |
| Depression | 12.50 (6.60) | 7.75 (5.99) |
| Generalized anxiety | 10.28 (5.93) | 6.85 (5.86) |

*Note*. Symptoms of depression were assessed with the 8-item Patient Health Questionnaire (Kroenke et al., 2001); symptoms of generalized anxiety disorders were measured with the 7-item General Anxiety Disorder Scale (Spitzer et al., 2006).

**Figure S1**

*Non-Significant Indirect Effect of Change in Loneliness on the Relationship Between Country and T3 Mental Health Problems*

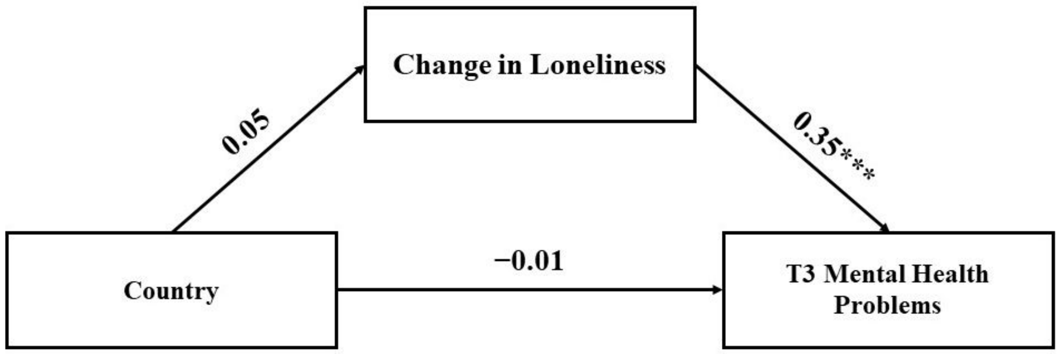


*Note.* Paths include standardized *B*s. * < .05, ** < 0.01, *** < 0.001. Mental health problems, COVID risk, and physical distancing adherence at T1, gender, and ethnicity were controlled for.

**Figure S2**

*Non-Significant Indirect Effect of Change in Interactions on the Relationship Between Country and T3 Mental Health Problems*

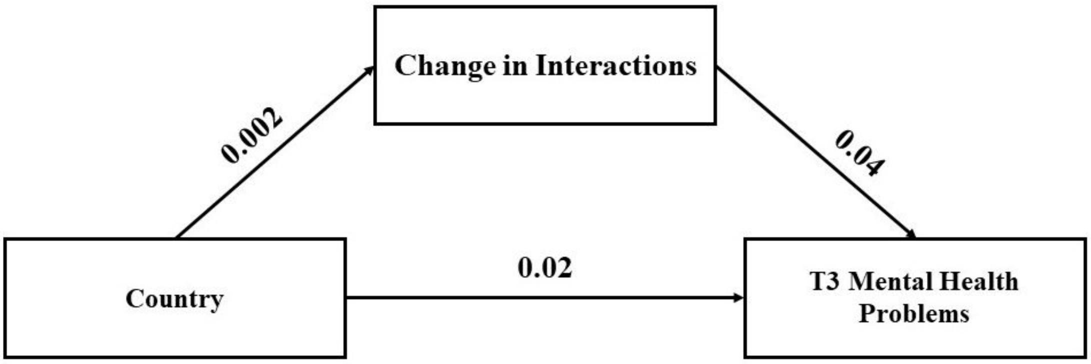


*Note.* Paths include standardized *B*s. * < .05, ** < 0.01, *** < 0.001. Mental health problems, COVID risk, and physical distancing adherence at T1, gender, and ethnicity were controlled for.

**Figure S3**

*Non-Significant Indirect Effect of Change in Social Support on the Relationship Between Country and T3 Mental Health Problems*

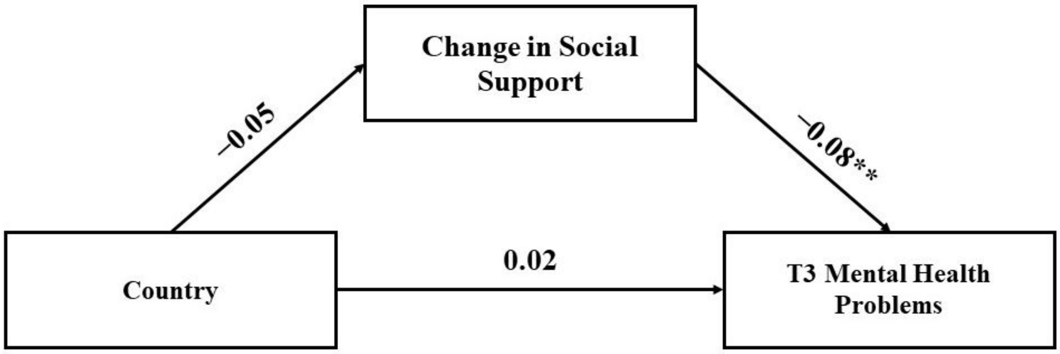


*Note.* Paths include standardized *B*s. * < .05, ** < 0.01, *** < 0.001. Mental health problems, COVID risk, and physical distancing adherence at T1, gender, and ethnicity were controlled for.

**Figure S4**

*Non-Significant Indirect Effect of Change in Loneliness on the Relationship Between Age and T3 Mental Health Problems*

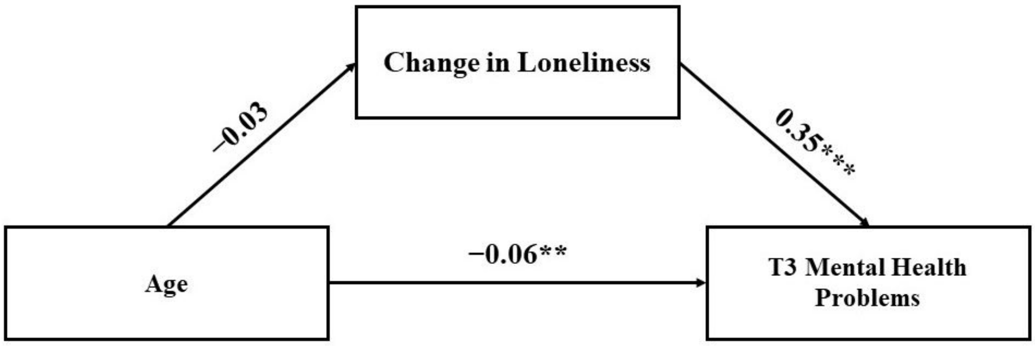


*Note.* Paths include standardized *B*s. * < .05, ** < 0.01, *** < 0.001. Mental health problems, COVID risk, and physical distancing adherence at T1, gender, and ethnicity were controlled for.

**Figure S5**

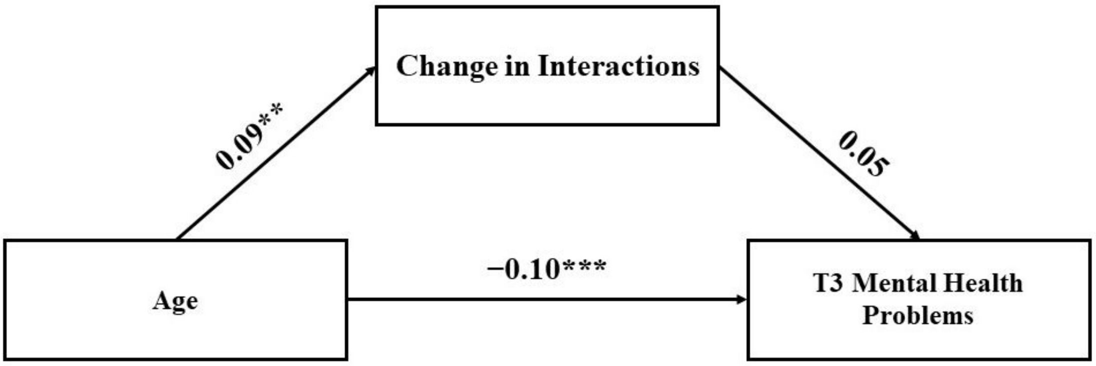


*Non-Significant Indirect Effect of Change in Interactions on the Relationship Between Age and T3 Mental Health Problems*

*Note.* Paths include standardized *B*s. * < .05, ** < 0.01, *** < 0.001. Mental health problems, COVID risk, and physical distancing adherence at T1, gender, and ethnicity were controlled for.

**Figure S6**

*Non-Significant Indirect Effect of Change in Social Support on the Relationship Between Age and T3 Mental Health Problems*

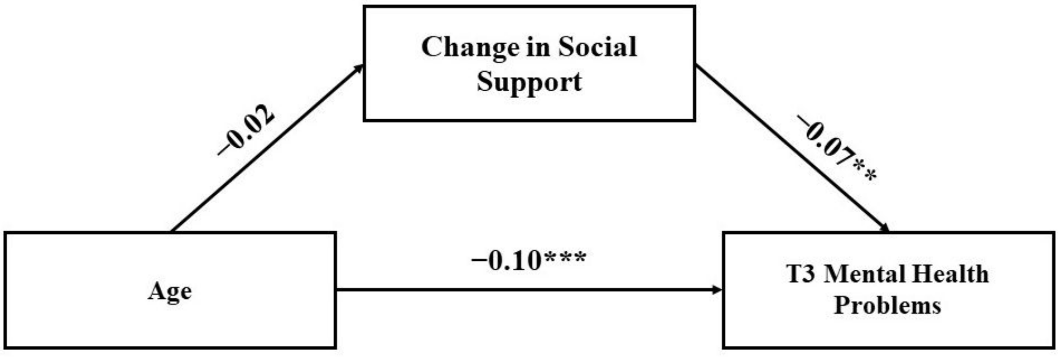


*Note.* Paths include standardized *B*s. * < .05, ** < 0.01, *** < 0.001. Mental health problems, COVID risk, and physical distancing adherence at T1, gender, and ethnicity were controlled for.

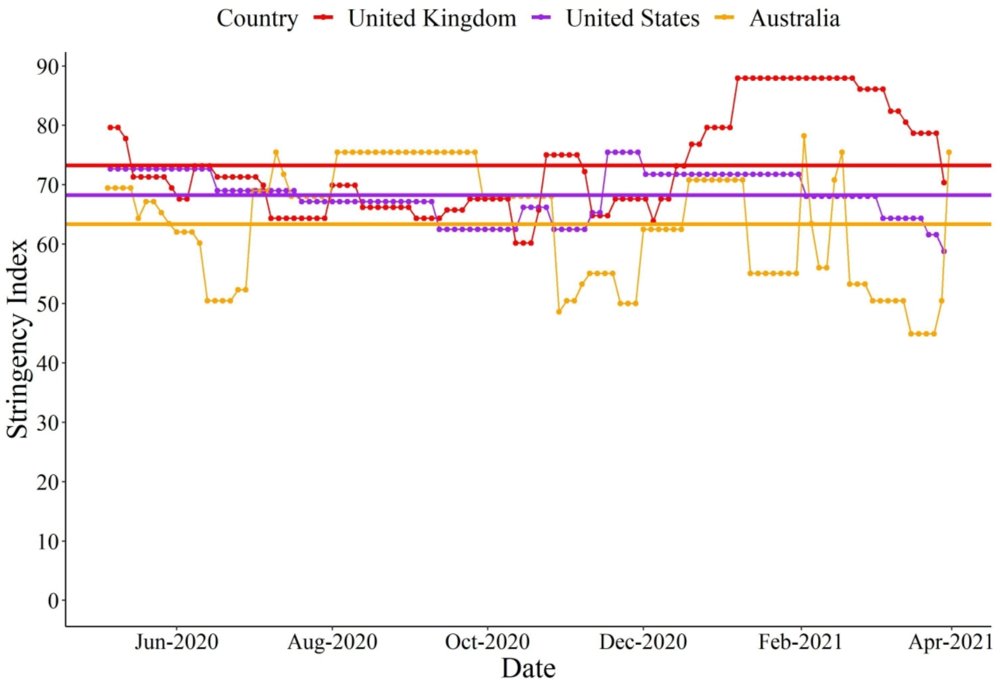


254x177mm (300 x 300 DPI)

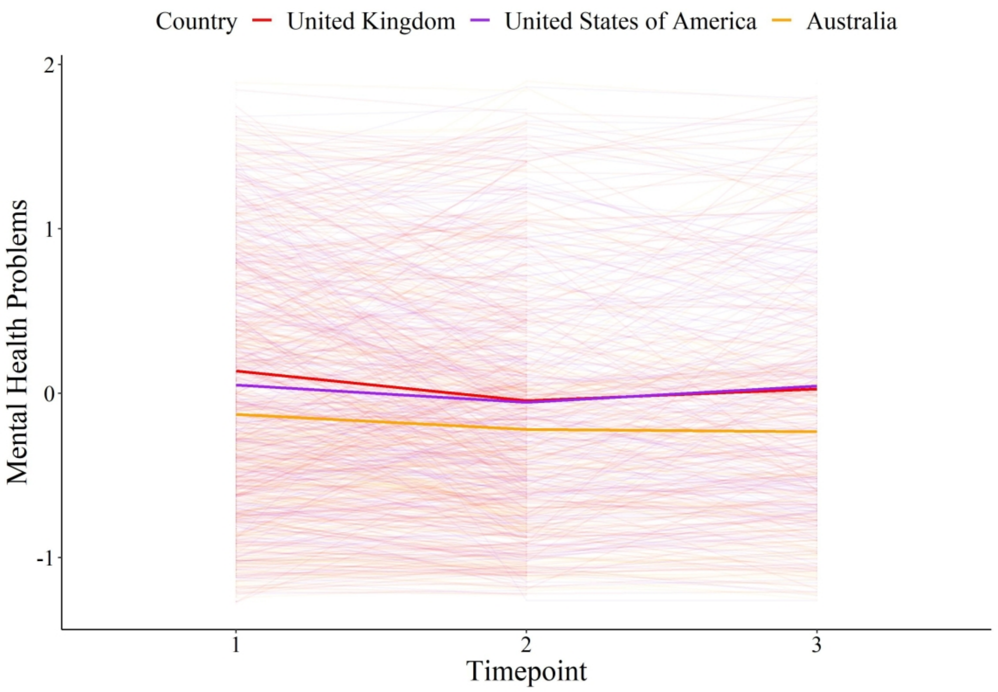


254x177mm (300 x 300 DPI)

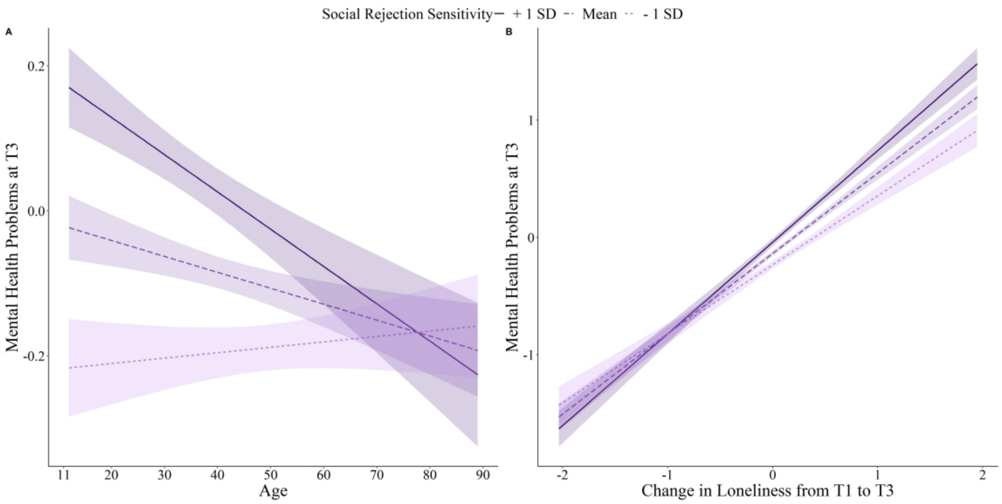


508x254mm (300 x 300 DPI)

**Table 1**

*Summary of Participant Characteristics*

| Participant Characteristics | *n* (%) |
|---|---|
| **Age** | |
| 11 – 24 years old | 436 (18.42%) |
| 25 – 64 years old | 1555 (65.69%) |
| 65 – 100 years old | 351 (14.83%) |
| Missing | 25 (1.06%) |
| **Gender Identity** | |
| Female | 2129 (89.95%) |
| Male | 211 (8.91%) |
| Other | 21 (0.89%) |
| Prefer not to say | 6 (0.25%) |
| **Country** | |
| United Kingdom | 1075 (45.42%) |
| United States of America | 699 (29.53%) |
| Australia | 593 (25.05%) |
| **Ethnicity** | |
| White | 1995 (84.32%) |
| Asian | 105 (4.44%) |
| Mixed | 71 (3%) |
| Hispanic | 44 (1.86%) |
| African | 16 (0.68%) |
| Aboriginal or Torres Strait Islander | 10 (0.42%) |

| | |
|---|---|
| Other | 95 (4.02%) |
| Prefer not to say | 30 (1.27%) |
| Missing | 1 (0.04%) |
| **SES** | |
| High | 1635 (69.07%) |
| Middle | 654 (27.63%) |
| Low | 5 (0.21%) |
| Missing | 73 (3.09%) |

*Note.* SES = Socioeconomic status. For participants over the age of 18 years, SES was operationalised as their highest educational attainment. For participants under the age of 18 years, SES was operationalised as the average of their parent's highest educational attainment; high = university, middle = high school or professional/vocational training, low = primary school.

**Table 2**

*Mental Health, Loneliness, Social Interactions, Social Support*

| | T1 | T2 | T3 |
|---|---|---|---|
| | ***n; M (SD)*** | ***n; M (SD)*** | ***n; M (SD)*** |
| **Clinical characteristics** | | | |
| Depressive symptoms | 2,366; 9.35 (6.32) | 825; 8.04 (6.27) | 743; 8.42 (6.32) |
| Anxiety symptoms | 2,352; 8.14 (6.18) | 822; 6.73 (5.83) | 743; 7.31 (6.00) |
| Mental well-being | 2,339; 19.64 (3.96) | 819; 20.25 (4.06) | 739; 19.88 (4.01) |
| Loneliness | 2,357; 25.65 (15.75) | 834; 25.66 (16.01) | 753; 27.49 (15.87) |
| **Social interactions** | | | |
| Face-to-face interactions | 2,089; 1.35 (0.66) | 851; 2.24 (1.12) | 744; 1.98 (1.02) |
| Video game interactions | 1,820; 2.51 (1.35) | 761; 3.26 (0.78) | 700; 3.25 (0.80) |
| Messaging interactions | 1,998; 3.02 (1.35) | 814; 3.59 (0.73) | 739; 3.62 (0.73) |
| Social media interactions | 2,019; 3.20 (1.38) | 816; 3.67 (0.80) | 750; 3.70 (0.81) |
| Video calling interactions | 2,024; 3.08 (1.48) | 822; 3.50 (0.92) | 731; 3.54 (0.95) |
| Voice calling interactions | 1,881; 2.86 (1.41) | 795; 3.38 (0.84) | 716; 3.35 (0.85) |
| **Social support** | | | |
| Social support from friends | 1,706; 2.75 (1.15) | 801; 2.95 (0.85) | 721; 2.86 (0.87) |
| Social support from family | 1,764; 3.11 (1.13) | 811; 3.10 (0.85) | 724; 3.05 (0.87) |

*Note*. Depressive symptoms measured with the 8-item Patient Health Questionnaire(Kroenke et al., 2001); Anxiety symptoms measured with the 7-item General Anxiety Disorder Scale(Spitzer et al., 2006); Mental well-being measured with the 7-item Warwick Edinburgh Mental Well-being Scale(Stewart-Brown et al., 2009); Loneliness measured with the 20-item UCLA Loneliness Scale(Russell et al., 1978); Face-to-face, video game, messaging, social media, video calling, and voice calling interactions = change in each type of interaction since

the start of the pandemic (T1) or since the previous time point (T2 and T3) measured on a visual analogue scale ranging from 1 – 5; Social support from friends and family = change in social support from friends and family since the start of the pandemic (T1) or since the previous time point (T2 and T3) measured on a visual analogue scale ranging from 1 – 5.

**Table 3**

*Multi-Group Latent Growth Curve Model Assessing the Impact of Age on Mental Health Problems Across One Year of the Pandemic*

| | *B* | *SE* | *z* | *p* |
|---|---|---|---|---|
| **Intercept** | | | | |
| UK | 0.32 | 0.12 | 2.77 | 0.006 |
| USA | 0.27 | 0.11 | 2.39 | 0.017 |
| Australia | 0.11 | 0.11 | 0.97 | 0.331 |
| **Slope** | 0.00 | 0.06 | 0.02 | 0.986 |
| **Regressions** | | | | |
| Intercept | | | | |
| Age | −0.01 | 0.001 | −12.60 | < 0.001 |
| Female | 0.03 | 0.06 | 0.63 | 0.529 |
| White | −0.06 | 0.04 | −1.40 | 0.161 |
| COVID-19 risk | 0.03 | 0.02 | 2.02 | 0.043 |
| Physical distancing adherence | 0.04 | 0.02 | 2.23 | 0.026 |
| Slope | | | | |
| Age | −0.00 | 0.00 | −1.17 | 0.244 |
| Female | −0.04 | 0.03 | −1.48 | 0.140 |
| White | 0.05 | 0.03 | 1.78 | 0.076 |
| COVID-19 risk | −0.01 | 0.01 | −1.60 | 0.110 |
| Physical distancing adherence | 0.00 | 0.01 | 0.03 | 0.974 |
| **Variances** | | | | |
| MH1 | 0.13 | 0.02 | 5.66 | < 0.001 |

| | | | | |
|---|---|---|---|---|
| MH2 | 0.10 | 0.01 | 10.34 | < 0.001 |
| MH3 | 0.06 | 0.03 | 2.56 | 0.011 |
| Intercept | 0.37 | 0.02 | 15.57 | < 0.001 |
| Slope | 0.01 | 0.01 | 1.09 | 0.276 |

*Note*. MH1 = mental health problems at T1, MH2 = mental health problems at T2, MH3 = mental health problems at T3. Mental health problems were modelled as a latent factor score comprising depression symptoms, measured with the 8-item Patient Health Questionnaire (Kroenke et al., 2001), anxiety symptoms, measured with the 7-item General Anxiety Disorder Scale (Spitzer et al., 2006), and mental wellbeing, measured with the 7-item Warwick Edinburgh Mental Wellbeing Scale (Stewart-Brown et al., 2009). COVID-19 risk was a composite score comprising a series of bespoke, binary items indexing whether participants had been quarantined or hospitalized due to COVID-19, or whether they knew anyone who had been diagnosed with, hospitalised or passed away from COVID-19. Physical distancing adherence was a bespoke item indexing the extent to which participants were complying with the physical distancing measures in place in their community, measured on a scale from 1 (*Not at all*) to 6 (*Extremely*). Due to large amounts of missing data on the COVID-19 risk and physical distancing adherence variables at T2 and T3, only T1 values of these variables were controlled for.
